# The complete chloroplast genome of *Aconitum austrokoreense* Koidz. (Ranunculaceae), an endangered endemic species in Korea

**DOI:** 10.1080/23802359.2016.1219644

**Published:** 2016-09-01

**Authors:** Ji-Eun Choi, Goon-Bo Kim, Chae-Eun Lim, Hee-Ju Yu, Jeong-Hwan Mun

**Affiliations:** a National Institute of Biological Resources, Incheon, Korea;; b Department of Bioscience and Bioinformatics, Myongji University, Yongin, Korea;; c Department of Life Science, The Catholic University of Korea, Bucheon, Korea

**Keywords:** *Aconitum austrokoreense*, endemic, chloroplast genome, sequence variation

## Abstract

We determined the complete chloroplast genome sequences of *Aconitum austrokoreense* Koidz., an endangered endemic species in Korea. The chloroplast DNA is 155,682 bp in length and encodes 37 tRNAs, 8 rRNAs, and 86 protein-coding genes. Phylogenetic analysis and sequence comparison of protein-coding genes with those in other Ranunculaceae chloroplast DNAs showed that the chloroplast genome of *A. austrokoreense* is closely related to that of *A. chiisanense* and large sequence variations identified in *rps16*, *matK*, and *rpl20* are specific to these two species.

*Aconitum austrokoreense* Koidz. is a member of genus *Aconitum* in Ranunculaceae and can be easily distinguished from other species in the genus by undivided or indistinctly 3-lobed leaves, yellowish white flowers with light violet margin, and pubescent pedicels and ovaries (Kadota 1987). *Aconitum austrokoreense* is an endemic species in Korea showing a very limited distribution with a few populations in rocky habitats near the trails along the mountain valleys (Kadota 1987; Park 2007). In Eastern Asia, the *Aconitum* species have been well known for their toxicity and medicinal effect (Lee 1979; Tang et al. 2012). In particular, the dried tuber of *A*. *austrokoreense* along with other *Aconitum* species has been commonly used as traditional herbal medicine to treat symptoms such as atonia, inflammation, paralysis, and coldness of the extremities (Lee et al. 1997). In recent years, natural *A*. *austrokoreense* populations continue to decline due to its limited distribution, over-harvesting for medicinal use, and habitat loss (Suh et al. 2004). For this reason, it is currently classified as a vulnerable species on the Red Data Book of Endangered Vascular Plants in Korea (Suh & Kim 2012) and has been protected as Level II Endangered Wild Plants by the Ministry of Environment, Korea.

In this study, we determined the complete chloroplast (cp) DNA sequence of *A*. *austrokoreense* that will be a valuable resource for genetic and phylogenetic study of this species. The specimen was collected from the valleys of Mt. Chiri and deposited in National Institute of Biological Resources with the accession number NIBR-VP0000494177. Sequencing was performed using the Illumina MiSeq platform (Illumina Inc., San Diego, CA) and a total of 28 million paired-end reads were generated and assembled into a circular DNA (Genbank accession KT820663). The cpDNA was 155,682 bp in length and consisted of two IRs (25,613 bp), one LSC (86,910 bp), and one SSC (17,546 bp). Annotation using DOGMA (Wyman et al. 2004) and Geneious (R7.1.8; Kearse et al. 2012) predicted a total of 131 genes (37 tRNA, 8 rRNA, and 86 protein-coding genes), of which 18 genes (7 tRNA, 4 rRNA, and 7 protein-coding genes) were duplicated into two IRs. A total of 22 genes (8 tRNA and 14 protein-coding genes) contained one or two introns. The maximum likelihood tree constructed using 79 non-redundant protein-coding genes in cpDNAs from 12 Ranunculaceae species and two outgroup species indicated that the cp genome of *A. austrokoreense* is closely related to the cp DNA of *A. chiisanense* that we reported recently (Lim et al. 2015; Figure 1). Furthermore, sequence comparison identified several distinct sequence variations in protein-coding genes of *Aconitum* species. A large deletion within *ycf2* and loss of *rpl32* were evident in all *Aconitum* species, whereas truncation of *rps16* and *matK* and insertion in *rpl20* were specific to *A. austrokoreense* and *A. chiisanense*. The unique sequence divergence identified in the cpDNA of *A. austrokoreense* will serve as a molecular basis for conservation and maintenance of this species as well as phylogenetic study of *Aconitum* and Ranunculaceae.

**Figure 1.**
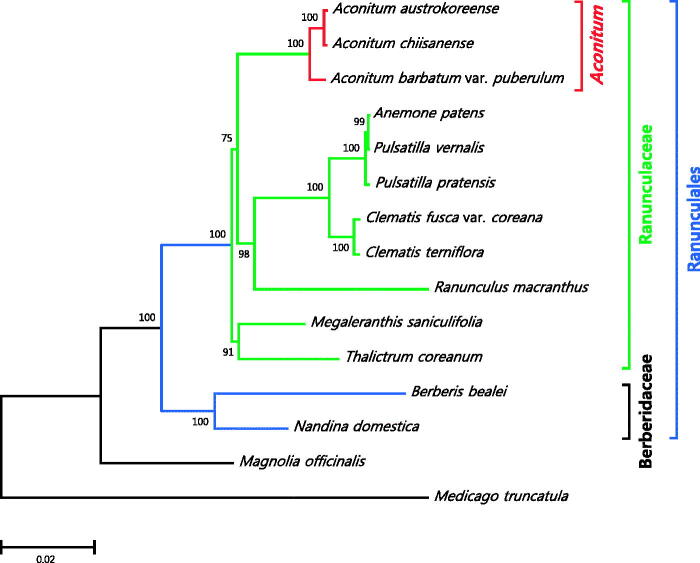
Maximum likelihood tree based on the chloroplast protein-coding genes of 15 taxa including *Aconitum austrokoreense*. Nucleotide sequences of 75 non-redundant conserved protein-coding genes from order Ranunculales as well as distantly related taxa were aligned and used for maximum likelihood phylogenetic analysis in MEGA7 with bootstrap test of 1000 replications. The NCBI accession numbers of chloroplast DNA sequences used in this study are as follows: *Aconitum austrokoreense* (KT820663), *A. barbatum* var. *puberulum* (KC844054), *A. chiisanense* (KT820665), *Anemone patens* (KR297057), *Berberis bealei* (NC022457), *Clematis fusca* var. *coreana* (KM652489), *C. terniflora* (NC028000), *Megaleranthis saniculifolia* (NC012615), *Nandina domestica* (NC008336), *Pulsatilla pratensis* (KR297059), *P. vernalis* (KR297061), *Ranunculus macranthus* (DQ359689), *Thalictrum coreanum* (KM206568), *Magnolia officinalis* (NC020316), and *Medicago truncatula* (NC003119). The scale bar represents the number of substitutions per site.
